# 4-Amino-*N*-(4,6-dimethyl­pyrimidin-2-yl)benzene­sulfonamide–2-nitro­benzoic acid (1/1)

**DOI:** 10.1107/S1600536813000779

**Published:** 2013-01-12

**Authors:** Graham Smith, Urs D. Wermuth

**Affiliations:** a Science and Engineering Faculty, Queensland University of Technology, GPO Box 2434, Brisbane, Queensland 4001, Australia

## Abstract

In the asymmetric unit of the title co-crystal, C_12_H_14_N_4_O_2_S·C_7_H_5_NO_4_, the sulfamethazine and 2-nitro­benzoic acid mol­ecules form a heterodimer through inter­molecular amide–carb­oxy­lic acid N—H⋯O and carb­oxy­lic acid–pyrimidine O—H⋯N hydrogen-bond pairs, giving a cyclic motif [graph set *R*_2_^2^(8)]. The dihedral angle between the two aromatic ring systems in the sulfamethazine mol­ecule is 88.96 (18)° and the nitro group of the acid is 50% rotationally disordered. Secondary aniline N—H⋯O_sulfone_ hydrogen-bonding associations give a two-dimensional structure lying parallel to the *ab* plane.

## Related literature

For background to sulfamethazole as a model for co-crystal formation, see: Caira (2007 ▶); Ghosh *et al.* (2011 ▶). For structures of 1:1 adducts of sulfamethazine with nitro­benzoic acid analogues, see: Lynch *et al.* (2000 ▶); Smith & Wermuth (2012 ▶). For graph-set analysis, see: Etter *et al.* (1990 ▶).
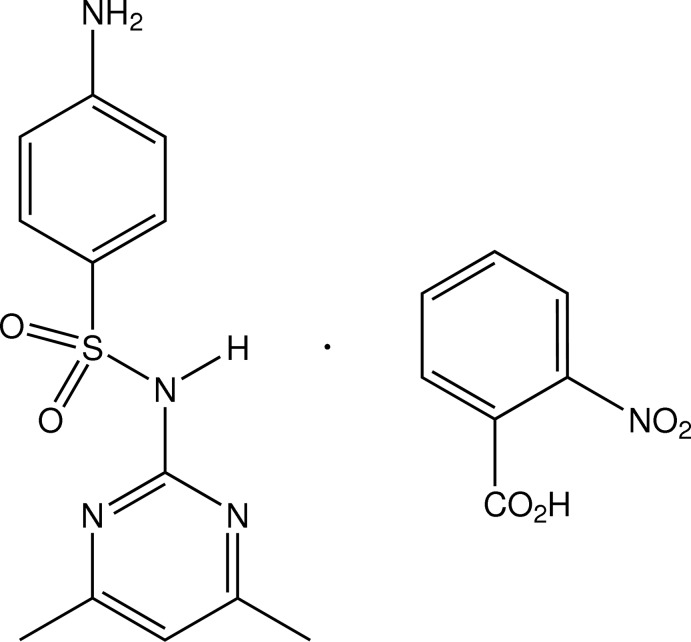


## Experimental

### 

#### Crystal data

C_12_H_14_N_4_O_2_S·C_7_H_5_NO_4_
*M* *_r_* = 445.46
Orthorhombic, 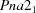

*a* = 14.2945 (4) Å
*b* = 8.0115 (3) Å
*c* = 19.0962 (5) Å
*V* = 2186.91 (12) Å^3^
*Z* = 4
Mo *K*α radiation
μ = 0.19 mm^−1^
*T* = 200 K
0.30 × 0.21 × 0.12 mm

#### Data collection

Oxford Diffraction Gemini-S CCD-detector diffractometer
Absorption correction: multi-scan (*CrysAlis PRO*; Agilent, 2012 ▶) *T*_min_ = 0.964, *T*_max_ = 0.980
5541 measured reflections
2777 independent reflections
2587 reflections with *I* > 2σ(*I*)
*R*_int_ = 0.023

#### Refinement

*R*[*F* ^2^ > 2σ(*F* ^2^)] = 0.040
*wR*(*F* ^2^) = 0.099
*S* = 1.04
2777 reflections
286 parameters
29 restraints
H-atom parameters constrained
Δρ_max_ = 0.29 e Å^−3^
Δρ_min_ = −0.21 e Å^−3^
Absolute structure: Flack (1983 ▶), 565 Friedel pairs
Flack parameter: 0.08 (9)

### 

Data collection: *CrysAlis PRO* (Agilent, 2012 ▶); cell refinement: *CrysAlis PRO*; data reduction: *CrysAlis PRO*; program(s) used to solve structure: *SIR92* (Altomare *et al.*, 1993 ▶); program(s) used to refine structure: *SHELXL97* (Sheldrick, 2008 ▶) within *WinGX* (Farrugia, 2012 ▶); molecular graphics: *PLATON* (Spek, 2009 ▶); software used to prepare material for publication: *PLATON*.

## Supplementary Material

Crystal structure: contains datablock(s) global, I. DOI: 10.1107/S1600536813000779/gg2106sup1.cif

Structure factors: contains datablock(s) I. DOI: 10.1107/S1600536813000779/gg2106Isup2.hkl

Supplementary material file. DOI: 10.1107/S1600536813000779/gg2106Isup3.cml

Additional supplementary materials:  crystallographic information; 3D view; checkCIF report

## Figures and Tables

**Table 1:** Hydrogen-bond geometry (Å, °)

*D*—H⋯*A*	*D*—H	H⋯*A*	*D*⋯*A*	*D*—H⋯*A*
O12—H12⋯N1*A*	0.90	1.77	2.671 (4)	180
N2*A*—H2*A*⋯O11	0.90	2.01	2.862 (4)	158
N41*A*—H41*A*⋯O11*A* ^i^	0.92	2.18	2.990 (3)	147
N41*A*—H42*A*⋯O12*A* ^ii^	0.83	2.24	2.973 (3)	146

Symmetry codes: (i) 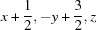; (ii) 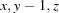.

**Fig. 1.**
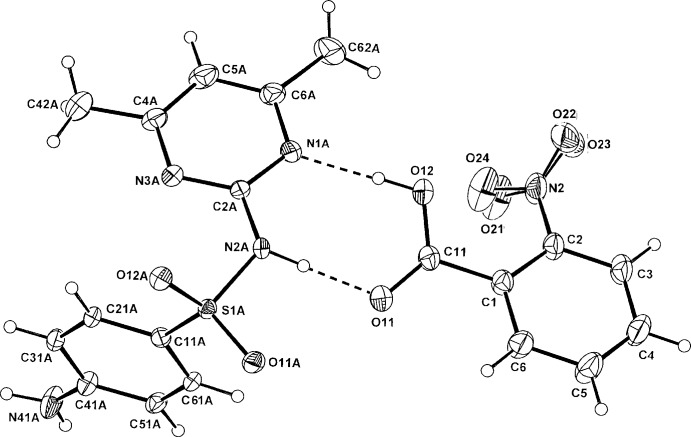
Molecular conformation and atom-numbering scheme for the title co-crystal, with inter-species hydrogen bonds shown as a dashed lines. The nitro group of the adduct molecule is 50% rotationally disordered and non-H atoms are shown as 30% probability displacement ellipsoids.

**Fig. 2.**
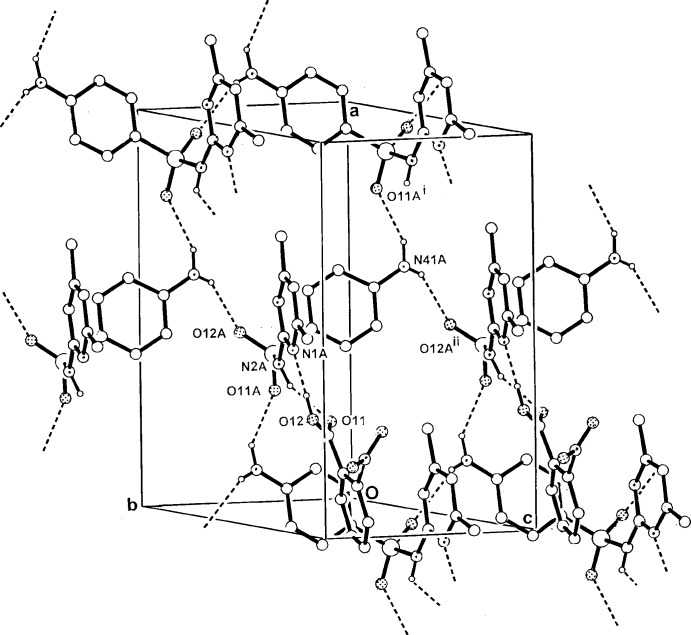
A perspective view of the two-dimensional structure which extends along [110], showing hydrogen-bonding associations as dashed lines, with the nitro group disorder not shown.

**Fig. 3.**
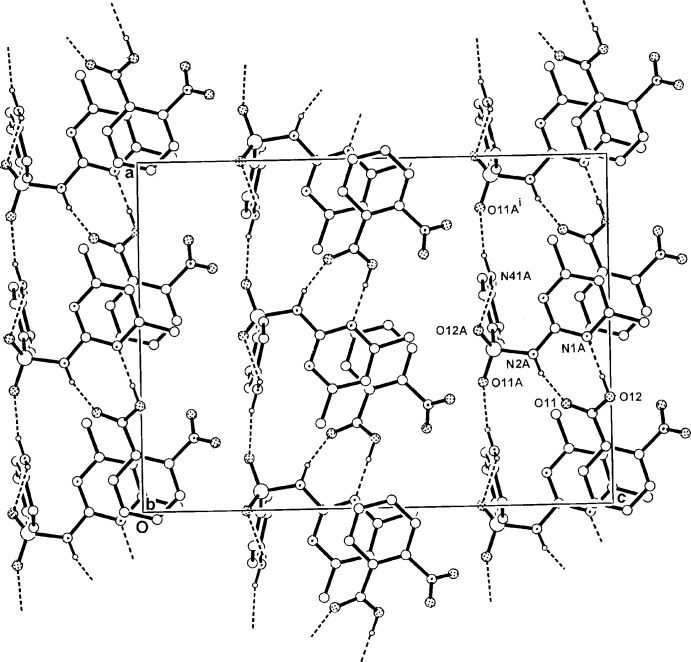
A view of the sheet structure along the b axis.

## supplementary crystallographic information

### Comment

The drug sulfamethazine [4-amino-*N*-(4,6-dimethylpyrimidin-2-yl)benzenesulfonamide] has been used as a model for co-crystal formation (Caira, 2007; Ghosh *et al.*, 2011)), commonly forming 1:1 adducts with carboxylic acids and amides, particularly the benzoic acid analogues. The structures of a number of these are known, including those with 4-nitrobenzoic acid (Smith & Wermuth, 2012) and 2,4-dinitrobenzoic acid (Lynch *et al.*, 2000). In these co-crystals, and in sulfamethazine adducts generally a common structural feature is the cyclic heterodimeric hydrogen-bonding association involving amide N—H···O_carboxyl_–carboxylic acid O—H···N_pyrimidine_ pairs [graph set *R*^2^_2_(8) (Etter *et al.*, 1990)].

Our 1:1 stoichiometric interaction of sulfamethazine with 2-nitrobenzoic acid gave the co-crystalline adduct C_12_H_14_N_4_O_2_S. C_7_H_5_NO_4_, the title compound and the structure is reported herein. In the sulfamethazine component (Fig. 1) the dihedral angle between the pyrimidine ring and the phenyl ring is 89.98 (18)° which compares with 82.33 (9)° and 78.77 (8)° for the two independent molecules in the 4-nitrobenzoic acid analogue (Smith & Wermuth, 2012). The angles between these two rings and the phenyl ring of the 2-nitrobenzoic acid molecule are 9.65 (19) and 88.22 (19)°, respectively. In the crystal the sulfamethazine and 2-nitrobenzoic acid molecules interact as previously described, giving cyclic *R*^2^_2_(8) hydrogen-bonded heterodimers (Table 1, Fig. 1).

Intermolecular amine N—H···O_sulfone_ hydrogen-bonding interactions link the heterodimer units along *a* (Fig. 2) as well as down *b*, forming two-dimensional sheet structures which extend along [110]. Unlike the isomeric 4-nitrobenzoic acid adduct there are no π–π interactions present in the structure but there are 52.2 Å^3^ potential solvent accessible voids present. The oxygen atoms of the nitro group of the adduct acid molecule are rotationally disordered over four 50% occupancy sites [O21, O22 and O23, O24]. In the absence of chirality in the molecules, the Flack absolute structure parameter [0.08 (9)] is of no structural significance.

### Experimental

The title compound was formed in the interaction of 1 mmol quantities of 4-amino-*N*-(4,6-dimethylpyrimidin-2-yl)benzenesulfonamide (sulfamethazine) and 2-nitrobenzoic acid in 50 ml of 50% ethanol–water with 10 min refluxing. Partial evaporation of the solvent gave a pale yellow solid which gave crystal plates suitable for the X-ray analysis after recrystallization from ethanol.

### Refinement

Hydrogen atoms potentially involved in hydrogen-bonding interactions were located by difference methods but were subsequently allowed to ride in the refinement with *U*_iso_(H) = 1.2*U*_eq_(N) or 1.5*U*_eq_(O). Other H atoms were included at calculated positions [C—H (aromatic) = 0.93 Å or C—H (methyl) = 0.96 Å] and also treated as riding, with *U*_iso_(H) = 1.2*U*_eq_(C) (aromatic) or 1.5*U*_eq_(C) (methyl). The nitro group was found to be rotationally disordered giving occupancies for the oxygen atoms O21, O22 [S.O.F. = 0.51 (1)] and O23, O24 [0.49 (1)] respectively and these were fixed at 0.50 in the final refinement cycles.

### Crystal data

**Table:**

C_12_H_14_N_4_O_2_S·C_7_H_5_NO_4_	*F*(000) = 928
*M**_r_* = 445.46	*D*_x_ = 1.353 Mg m^−^^3^
Orthorhombic, *P**n**a*2_1_	Mo *K*α radiation, λ = 0.71073 Å
Hall symbol: P 2c -2n	Cell parameters from 3011 reflections
*a* = 14.2945 (4) Å	θ = 3.1–28.8°
*b* = 8.0115 (3) Å	µ = 0.19 mm^−^^1^
*c* = 19.0962 (5) Å	*T* = 200 K
*V* = 2186.91 (12) Å^3^	Plate, yellow
*Z* = 4	0.30 × 0.21 × 0.12 mm

### Data collection

**Table:**

Oxford Diffraction Gemini-S CCD-detector diffractometer	2777 independent reflections
Radiation source: Enhance (Mo) X-ray source	2587 reflections with *I* > 2σ(*I*)
Graphite monochromator	*R*_int_ = 0.023
Detector resolution: 16.077 pixels mm^-1^	θ_max_ = 26.0°, θ_min_ = 3.1°
ω scans	*h* = −17→9
Absorption correction: multi-scan (*CrysAlis PRO*; Agilent, 2012)	*k* = −8→9
*T*_min_ = 0.964, *T*_max_ = 0.980	*l* = −23→8
5541 measured reflections	

### Refinement

**Table:**

Refinement on *F*^2^	Secondary atom site location: difference Fourier map
Least-squares matrix: full	Hydrogen site location: inferred from neighbouring sites
*R*[*F*^2^ > 2σ(*F*^2^)] = 0.040	H-atom parameters constrained
*wR*(*F*^2^) = 0.099	*w* = 1/[σ^2^(*F*_o_^2^) + (0.0555*P*)^2^ + 0.5356*P*] where *P* = (*F*_o_^2^ + 2*F*_c_^2^)/3
*S* = 1.04	(Δ/σ)_max_ = 0.001
2777 reflections	Δρ_max_ = 0.29 e Å^−^^3^
286 parameters	Δρ_min_ = −0.21 e Å^−^^3^
29 restraints	Absolute structure: Flack (1983), 565 Friedel pairs
Primary atom site location: structure-invariant direct methods	Flack parameter: 0.08 (9)

### Special details

**Table:**

Geometry. Bond distances, angles *etc*. have been calculated using the rounded fractional coordinates. All su's are estimated from the variances of the (full) variance-covariance matrix. The cell e.s.d.'s are taken into account in the estimation of distances, angles and torsion angles

### Fractional atomic coordinates and isotropic or equivalent isotropic displacement parameters (Å^2^)

**Table:**

	*x*	*y*	*z*	*U*_iso_*/*U*_eq_	Occ. (<1)
S1A	0.44672 (4)	1.02307 (8)	0.75273 (5)	0.0249 (2)	
O11A	0.35295 (13)	0.9968 (3)	0.72869 (13)	0.0342 (7)	
O12A	0.50151 (14)	1.1513 (3)	0.72083 (12)	0.0326 (7)	
N1A	0.47662 (18)	1.1033 (3)	0.95152 (15)	0.0352 (8)	
N2A	0.43078 (16)	1.0714 (3)	0.83654 (14)	0.0288 (8)	
N3A	0.59047 (17)	1.0898 (3)	0.86027 (15)	0.0334 (8)	
N41A	0.65507 (17)	0.3924 (3)	0.7489 (2)	0.0538 (12)	
C2A	0.5032 (2)	1.0887 (4)	0.88442 (16)	0.0273 (9)	
C4A	0.6585 (2)	1.1028 (4)	0.9085 (2)	0.0392 (11)	
C5A	0.6372 (3)	1.1216 (5)	0.9787 (2)	0.0497 (14)	
C6A	0.5446 (3)	1.1221 (5)	0.99900 (19)	0.0429 (11)	
C11A	0.50792 (17)	0.8370 (3)	0.75032 (18)	0.0262 (8)	
C21A	0.60295 (19)	0.8371 (4)	0.73347 (16)	0.0286 (9)	
C31A	0.6509 (2)	0.6892 (4)	0.73148 (18)	0.0312 (9)	
C41A	0.60616 (19)	0.5374 (3)	0.7475 (2)	0.0324 (9)	
C42A	0.7565 (2)	1.0928 (6)	0.8819 (3)	0.0592 (15)	
C51A	0.5098 (2)	0.5407 (4)	0.76412 (18)	0.0323 (9)	
C61A	0.46169 (19)	0.6885 (4)	0.76475 (17)	0.0289 (9)	
C62A	0.5143 (3)	1.1416 (7)	1.0737 (2)	0.0663 (16)	
O11	0.28633 (18)	0.8862 (4)	0.90536 (17)	0.0639 (10)	
O12	0.30259 (17)	1.0522 (4)	0.99775 (15)	0.0505 (9)	
O21	0.2682 (14)	0.800 (3)	1.1121 (18)	0.083 (5)	0.500
O22	0.1915 (11)	1.0471 (16)	1.1479 (5)	0.095 (4)	0.500
O23	0.1968 (11)	0.9621 (18)	1.1692 (5)	0.095 (4)	0.500
O24	0.2837 (14)	0.842 (3)	1.1026 (18)	0.083 (5)	0.500
N2	0.2084 (2)	0.9099 (6)	1.1124 (2)	0.0668 (14)	
C1	0.1619 (2)	0.9013 (4)	0.98691 (19)	0.0378 (10)	
C2	0.1370 (2)	0.8973 (5)	1.05718 (19)	0.0408 (11)	
C3	0.0460 (2)	0.8689 (5)	1.0792 (2)	0.0503 (14)	
C4	−0.0216 (3)	0.8434 (6)	1.0290 (3)	0.0613 (16)	
C5	0.0011 (3)	0.8421 (7)	0.9596 (3)	0.0680 (18)	
C6	0.0926 (3)	0.8725 (6)	0.9385 (2)	0.0580 (14)	
C11	0.2569 (2)	0.9455 (5)	0.95931 (19)	0.0387 (11)	
H2A	0.37560	1.03330	0.85240	0.0350*	
H5A	0.68450	1.13390	1.01170	0.0600*	
H21A	0.63350	0.93690	0.72370	0.0340*	
H31A	0.71390	0.68900	0.71940	0.0380*	
H41A	0.71650	0.38280	0.73590	0.0650*	
H42A	0.62490	0.30460	0.75470	0.0650*	
H43A	0.78730	0.99840	0.90250	0.0890*	
H44A	0.78950	1.19300	0.89420	0.0890*	
H45A	0.75570	1.08070	0.83190	0.0890*	
H51A	0.47880	0.44180	0.77470	0.0390*	
H61A	0.39800	0.68950	0.77480	0.0350*	
H62A	0.44730	1.13730	1.07620	0.0990*	
H63A	0.53580	1.24710	1.09130	0.0990*	
H64A	0.54040	1.05310	1.10130	0.0990*	
H3	0.03110	0.86720	1.12660	0.0600*	
H4	−0.08350	0.82690	1.04260	0.0740*	
H5	−0.04480	0.82070	0.92630	0.0820*	
H6	0.10710	0.87350	0.89100	0.0690*	
H12	0.36150	1.06900	0.98230	0.0760*	

### Atomic displacement parameters (Å^2^)

**Table:**

	*U*^11^	*U*^22^	*U*^33^	*U*^12^	*U*^13^	*U*^23^
S1A	0.0215 (3)	0.0274 (3)	0.0259 (3)	0.0012 (2)	−0.0003 (3)	−0.0001 (4)
O11A	0.0246 (10)	0.0395 (11)	0.0385 (13)	0.0037 (8)	−0.0055 (10)	−0.0031 (10)
O12A	0.0320 (11)	0.0301 (11)	0.0357 (12)	−0.0002 (8)	0.0019 (10)	0.0056 (11)
N1A	0.0352 (14)	0.0415 (16)	0.0289 (14)	−0.0063 (12)	0.0044 (12)	−0.0024 (13)
N2A	0.0196 (12)	0.0357 (13)	0.0312 (14)	−0.0001 (10)	0.0047 (11)	−0.0042 (12)
N3A	0.0287 (13)	0.0381 (15)	0.0334 (15)	−0.0045 (11)	0.0021 (12)	−0.0023 (13)
N41A	0.0292 (12)	0.0281 (13)	0.104 (3)	0.0001 (10)	0.0144 (19)	0.000 (2)
C2A	0.0290 (15)	0.0261 (15)	0.0267 (16)	−0.0033 (12)	0.0013 (13)	−0.0014 (13)
C4A	0.0333 (17)	0.0414 (18)	0.043 (2)	−0.0065 (14)	−0.0031 (17)	−0.0012 (17)
C5A	0.047 (2)	0.063 (3)	0.039 (2)	−0.0157 (18)	−0.0120 (18)	0.003 (2)
C6A	0.048 (2)	0.050 (2)	0.0306 (18)	−0.0159 (17)	0.0001 (16)	−0.0002 (17)
C11A	0.0234 (12)	0.0310 (14)	0.0242 (13)	0.0008 (10)	−0.0014 (14)	−0.0014 (15)
C21A	0.0236 (13)	0.0308 (14)	0.0314 (17)	−0.0061 (11)	0.0053 (12)	0.0009 (13)
C31A	0.0209 (13)	0.0333 (15)	0.0395 (19)	0.0001 (11)	0.0049 (13)	−0.0029 (14)
C41A	0.0264 (13)	0.0318 (14)	0.0391 (17)	−0.0005 (11)	0.0013 (17)	0.0005 (17)
C42A	0.0316 (19)	0.079 (3)	0.067 (3)	−0.007 (2)	−0.006 (2)	−0.008 (3)
C51A	0.0278 (14)	0.0282 (14)	0.041 (2)	−0.0072 (11)	0.0010 (15)	−0.0015 (15)
C61A	0.0190 (12)	0.0346 (15)	0.033 (2)	−0.0056 (10)	0.0014 (13)	−0.0021 (14)
C62A	0.080 (3)	0.090 (3)	0.029 (2)	−0.017 (3)	0.001 (2)	−0.004 (2)
O11	0.0497 (15)	0.092 (2)	0.0499 (18)	−0.0238 (14)	0.0210 (14)	−0.0240 (17)
O12	0.0360 (13)	0.0713 (17)	0.0443 (16)	−0.0111 (12)	0.0148 (12)	−0.0126 (15)
O21	0.054 (6)	0.123 (10)	0.073 (8)	0.001 (7)	−0.004 (4)	0.065 (6)
O22	0.060 (3)	0.186 (12)	0.039 (5)	−0.011 (7)	0.000 (5)	−0.033 (6)
O23	0.060 (3)	0.186 (12)	0.039 (5)	−0.011 (7)	0.000 (5)	−0.033 (6)
O24	0.054 (6)	0.123 (10)	0.073 (8)	0.001 (7)	−0.004 (4)	0.065 (6)
N2	0.0385 (19)	0.125 (3)	0.037 (2)	−0.011 (2)	0.0077 (17)	0.001 (2)
C1	0.0342 (16)	0.046 (2)	0.0333 (18)	−0.0038 (14)	0.0065 (16)	0.0044 (17)
C2	0.0335 (17)	0.052 (2)	0.037 (2)	−0.0019 (15)	0.0062 (16)	0.0041 (17)
C3	0.043 (2)	0.070 (3)	0.038 (2)	−0.0094 (18)	0.0142 (18)	0.003 (2)
C4	0.036 (2)	0.088 (3)	0.060 (3)	−0.020 (2)	0.010 (2)	−0.001 (3)
C5	0.042 (2)	0.113 (4)	0.049 (3)	−0.023 (2)	−0.008 (2)	0.006 (3)
C6	0.043 (2)	0.096 (3)	0.035 (2)	−0.015 (2)	0.0038 (17)	0.005 (2)
C11	0.0347 (18)	0.048 (2)	0.0335 (19)	−0.0029 (15)	0.0057 (16)	−0.0002 (17)

### Geometric parameters (Å, º)

**Table:**

S1A—O11A	1.432 (2)	C21A—C31A	1.369 (4)
S1A—O12A	1.428 (2)	C31A—C41A	1.408 (4)
S1A—N2A	1.662 (3)	C41A—C51A	1.414 (4)
S1A—C11A	1.729 (2)	C51A—C61A	1.369 (4)
O11—C11	1.210 (5)	C5A—H5A	0.9300
O12—C11	1.302 (5)	C21A—H21A	0.9300
O21—N2	1.23 (2)	C31A—H31A	0.9300
O22—N2	1.314 (13)	C42A—H45A	0.9600
O23—N2	1.174 (11)	C42A—H43A	0.9600
O24—N2	1.22 (2)	C42A—H44A	0.9600
O12—H12	0.9000	C51A—H51A	0.9300
N1A—C6A	1.338 (5)	C61A—H61A	0.9300
N1A—C2A	1.342 (4)	C62A—H62A	0.9600
N2A—C2A	1.388 (4)	C62A—H63A	0.9600
N3A—C2A	1.330 (4)	C62A—H64A	0.9600
N3A—C4A	1.343 (4)	C1—C6	1.375 (5)
N41A—C41A	1.356 (3)	C1—C11	1.499 (4)
N2A—H2A	0.9000	C1—C2	1.389 (5)
N41A—H41A	0.9200	C2—C3	1.386 (4)
N41A—H42A	0.8300	C3—C4	1.376 (6)
N2—C2	1.471 (5)	C4—C5	1.365 (8)
C4A—C42A	1.492 (4)	C5—C6	1.390 (6)
C4A—C5A	1.383 (5)	C3—H3	0.9300
C5A—C6A	1.379 (6)	C4—H4	0.9300
C6A—C62A	1.499 (5)	C5—H5	0.9300
C11A—C21A	1.396 (4)	C6—H6	0.9300
C11A—C61A	1.389 (4)		
			
O11A—S1A—O12A	118.83 (14)	C11A—C21A—H21A	120.00
O11A—S1A—N2A	102.39 (13)	C31A—C21A—H21A	120.00
O11A—S1A—C11A	109.77 (13)	C21A—C31A—H31A	120.00
O12A—S1A—N2A	108.56 (13)	C41A—C31A—H31A	120.00
O12A—S1A—C11A	109.35 (13)	H43A—C42A—H44A	109.00
N2A—S1A—C11A	107.21 (15)	C4A—C42A—H44A	109.00
C2A—N1A—C6A	116.8 (3)	C4A—C42A—H43A	109.00
S1A—N2A—C2A	123.7 (2)	H44A—C42A—H45A	109.00
C2A—N3A—C4A	116.2 (3)	H43A—C42A—H45A	109.00
C2A—N2A—H2A	118.00	C4A—C42A—H45A	109.00
S1A—N2A—H2A	111.00	C61A—C51A—H51A	120.00
C41A—N41A—H41A	124.00	C41A—C51A—H51A	120.00
H41A—N41A—H42A	118.00	C11A—C61A—H61A	120.00
C41A—N41A—H42A	117.00	C51A—C61A—H61A	120.00
O24—N2—C2	118.1 (16)	H63A—C62A—H64A	110.00
O23—N2—C2	126.1 (8)	C6A—C62A—H62A	109.00
O21—N2—O22	137.0 (16)	C6A—C62A—H63A	109.00
O21—N2—C2	115.5 (15)	H62A—C62A—H63A	109.00
O22—N2—C2	107.4 (7)	H62A—C62A—H64A	110.00
N1A—C2A—N3A	126.6 (3)	C6A—C62A—H64A	109.00
N1A—C2A—N2A	115.3 (3)	C2—C1—C11	125.3 (3)
N2A—C2A—N3A	118.2 (3)	C6—C1—C11	117.1 (3)
N3A—C4A—C5A	120.9 (3)	C2—C1—C6	117.5 (3)
C5A—C4A—C42A	122.9 (4)	N2—C2—C3	116.4 (3)
N3A—C4A—C42A	116.2 (4)	C1—C2—C3	122.5 (3)
C4A—C5A—C6A	119.0 (4)	N2—C2—C1	120.9 (3)
N1A—C6A—C5A	120.4 (3)	C2—C3—C4	118.2 (4)
N1A—C6A—C62A	116.6 (4)	C3—C4—C5	120.7 (4)
C5A—C6A—C62A	123.0 (4)	C4—C5—C6	120.3 (4)
C21A—C11A—C61A	120.6 (2)	C1—C6—C5	120.8 (4)
S1A—C11A—C61A	119.5 (2)	O11—C11—C1	121.4 (3)
S1A—C11A—C21A	119.9 (2)	O12—C11—C1	114.3 (3)
C11A—C21A—C31A	119.5 (3)	O11—C11—O12	124.3 (3)
C21A—C31A—C41A	120.9 (3)	C2—C3—H3	121.00
C31A—C41A—C51A	118.4 (2)	C4—C3—H3	121.00
N41A—C41A—C31A	120.7 (3)	C3—C4—H4	120.00
N41A—C41A—C51A	120.9 (3)	C5—C4—H4	120.00
C41A—C51A—C61A	120.5 (3)	C4—C5—H5	120.00
C11A—C61A—C51A	120.0 (3)	C6—C5—H5	120.00
C4A—C5A—H5A	121.00	C1—C6—H6	120.00
C6A—C5A—H5A	120.00	C5—C6—H6	120.00
			
O11A—S1A—N2A—C2A	172.1 (2)	O23—N2—C2—C3	−31.7 (11)
O12A—S1A—N2A—C2A	−61.5 (3)	O24—N2—C2—C1	−36.8 (15)
C11A—S1A—N2A—C2A	56.6 (3)	O24—N2—C2—C3	138.5 (14)
O11A—S1A—C11A—C21A	145.0 (3)	O22—N2—C2—C3	−67.6 (7)
O11A—S1A—C11A—C61A	−34.6 (3)	O23—N2—C2—C1	153.1 (9)
O12A—S1A—C11A—C21A	13.0 (3)	C42A—C4A—C5A—C6A	−177.1 (4)
O12A—S1A—C11A—C61A	−166.6 (3)	N3A—C4A—C5A—C6A	1.8 (5)
N2A—S1A—C11A—C21A	−104.5 (3)	C4A—C5A—C6A—N1A	0.5 (6)
N2A—S1A—C11A—C61A	75.9 (3)	C4A—C5A—C6A—C62A	180.0 (4)
O24—O21—N2—O23	−106 (6)	C61A—C11A—C21A—C31A	−0.3 (5)
O24—O21—N2—C2	102 (6)	S1A—C11A—C61A—C51A	−178.9 (3)
O24—O21—N2—O22	−74 (7)	S1A—C11A—C21A—C31A	−179.8 (3)
O23—O22—N2—C2	127.6 (15)	C21A—C11A—C61A—C51A	1.5 (5)
O23—O22—N2—O21	−56 (3)	C11A—C21A—C31A—C41A	−1.2 (5)
O23—O22—N2—O24	−82 (3)	C21A—C31A—C41A—N41A	−176.6 (3)
O22—O23—N2—C2	−69.3 (18)	C21A—C31A—C41A—C51A	1.5 (5)
O22—O23—N2—O24	120 (2)	C31A—C41A—C51A—C61A	−0.2 (5)
O22—O23—N2—O21	142.5 (18)	N41A—C41A—C51A—C61A	177.9 (3)
O21—O24—N2—O22	124 (5)	C41A—C51A—C61A—C11A	−1.3 (5)
O21—O24—N2—O23	83 (6)	C6—C1—C2—N2	173.8 (4)
O21—O24—N2—C2	−88 (6)	C6—C1—C2—C3	−1.2 (6)
C2A—N1A—C6A—C62A	178.9 (4)	C11—C1—C2—N2	−10.5 (6)
C6A—N1A—C2A—N2A	−179.3 (3)	C11—C1—C2—C3	174.5 (4)
C6A—N1A—C2A—N3A	0.5 (5)	C2—C1—C6—C5	0.6 (6)
C2A—N1A—C6A—C5A	−1.7 (5)	C11—C1—C6—C5	−175.6 (4)
S1A—N2A—C2A—N1A	−170.5 (2)	C2—C1—C11—O11	149.7 (4)
S1A—N2A—C2A—N3A	9.7 (4)	C2—C1—C11—O12	−30.9 (5)
C4A—N3A—C2A—N2A	−178.5 (3)	C6—C1—C11—O11	−34.5 (5)
C2A—N3A—C4A—C5A	−2.9 (5)	C6—C1—C11—O12	144.8 (4)
C4A—N3A—C2A—N1A	1.8 (5)	N2—C2—C3—C4	−175.0 (4)
C2A—N3A—C4A—C42A	176.1 (3)	C1—C2—C3—C4	0.2 (6)
O21—N2—C2—C3	115.3 (15)	C2—C3—C4—C5	1.6 (7)
O22—N2—C2—C1	117.1 (7)	C3—C4—C5—C6	−2.3 (8)
O21—N2—C2—C1	−60.0 (15)	C4—C5—C6—C1	1.2 (8)

### Hydrogen-bond geometry (Å, º)

**Table:**

*D*—H···*A*	*D*—H	H···*A*	*D*···*A*	*D*—H···*A*
O12—H12···N1*A*	0.90	1.77	2.671 (4)	180
N2*A*—H2*A*···O11	0.90	2.01	2.862 (4)	158
N41*A*—H41*A*···O11*A*^i^	0.92	2.18	2.990 (3)	147
N41*A*—H42*A*···O12*A*^ii^	0.83	2.24	2.973 (3)	146
C3—H3···O12*A*^iii^	0.93	2.54	3.289 (4)	138
C21*A*—H21*A*···O12*A*	0.93	2.55	2.915 (4)	104
C31*A*—H31*A*···O11*A*^i^	0.93	2.49	3.250 (4)	139
C51*A*—H51*A*···O12*A*^ii^	0.93	2.57	3.230 (4)	129

Symmetry codes: (i) *x*+1/2, −*y*+3/2, *z*; (ii) *x*, *y*−1, *z*; (iii) −*x*+1/2, *y*−1/2, *z*+1/2.
